# Winter Climate Limits Subantarctic Low Forest Growth and Establishment

**DOI:** 10.1371/journal.pone.0093241

**Published:** 2014-04-01

**Authors:** Melanie A. Harsch, Matt S. McGlone, Janet M. Wilmshurst

**Affiliations:** 1 Department of Biology, University of Washington, Seattle, Washington, United States of America; 2 Ecosystems and Global Change, Landcare Research, Lincoln, New Zealand; WSL Institute for Snow and Avalanche Research SLF, Switzerland

## Abstract

Campbell Island, an isolated island 600 km south of New Zealand mainland (52°S, 169°E) is oceanic (Conrad Index of Continentality  = −5) with small differences between mean summer and winter temperatures. Previous work established the unexpected result that a mean annual climate warming of *c*. 0.6°C since the 1940's has not led to upward movement of the forest limit. Here we explore the relative importance of summer and winter climatic conditions on growth and age-class structure of the treeline forming species, *Dracophyllum longifolium* and *Dracophyllum scoparium* over the second half of the 20^th^ century. The relationship between climate and growth and establishment were evaluated using standard dendroecological methods and local climate data from a meteorological station on the island. Growth and establishment were correlated against climate variables and further evaluated within hierarchical regression models to take into account the effect of plot level variables. Winter climatic conditions exerted a greater effect on growth and establishment than summer climatic conditions. Establishment is maximized under warm (mean winter temperatures >7 °C), dry winters (total winter precipitation <400 mm). Growth, on the other hand, is adversely affected by wide winter temperature ranges and increased rainfall. The contrasting effect of winter warmth on growth and establishment suggests that winter temperature affects growth and establishment through differing mechanisms. We propose that milder winters enhance survival of seedlings and, therefore, recruitment, but increases metabolic stress on established plants, resulting in lower growth rates. Future winter warming may therefore have complex effects on plant growth and establishment globally.

## Introduction

Climate change has altered current plant distributions and demographic rates [1] and, based on model projections, is predicted to result in further, more extensive changes [2], [3], [4]. However, many species worldwide have not responded to recent climate warming by increasing elevation or latitude as would be expected if growing season temperature alone were limiting growth and distribution [5], [6], [7], [8].

Although there is no shortage of credible explanations for why some species have failed to respond in the expected manner, including recruitment limitations through sporadic seed set, competition from lower-growing plants, and unfavorable edaphic conditions above their current distribution, winter conditions appear to have been relatively neglected as an explanation [9]. This is understandable. For many years winter climate was a leading candidate for explaining many features of physiology, morphology and community structure of trees at their upper distributional limit [10], [11], [12]. However, most plant species at high elevations or latitudes have excellent cold resistance, and are either dormant during winter or insulated from harsh winter conditions by snow. Nevertheless, winters can affect plant growth at treeline through snow-pack duration and depth, frost, and wind-abrasion of leaves and stems. These factors destroy biomass and reduce the photosynthetic capacity of leaves and needles in evergreens (reviewed in Harsch and Bader [6]). It follows that warmer winter would exert a positive influence on plants, as has been observed with tree stem development [13] and treeline elevational expansion [7].

Negative effects of winter warmth on growth and establishment have been largely overlooked [14]. Winter warmth increases metabolic stress, alters soil processes, and dehardens foliage, reducing growth and preventing forest expansion [14], [15], [16]. The effect of winter warming is therefore likely to be complex. Winter warming has affected plant growth and survival, largely through changes in snowpack depth and duration [17], [18]. As annual snowpack levels are declining, it is important to assess how winter warming is affecting plants in cold environments with limited snowpack.

Here we use climate and dendrochronological data from a subantarctic island to show that winter climatic conditions are critical in determining growth rates and establishment of the dominant tree species. Uninhabited Campbell Island offers several advantages as a study site. It is at the southern limit to trees in this sector of the Pacific Ocean with a simple treeline community of one dominant genus (*Dracophyllum*). As its winters are warm (mean winter temperature: 4.9 °C) and snow lie is ephemeral, the site provides a case study for understanding plant response to warmer, less snowy winters. Anthropogenic disturbance (fire and farming) occurred between 1810 and 1931 but there has been no disturbance since.

The island has a long-established meteorological station (since 1941) [5], [8]. Treeline altitude is expected to have shifted by *c*. 50 m, based on observed annual climate warming since the 1940's (*c*. 0.6 °C, Fig. S1 in File S1). However, there has been no upward shift in treeline elevation from the positions recorded in the late 19^th^ century [19] despite rapid regeneration in cleared areas below treeline since the absence of any significant anthropogenic disturbance [8]. We present climate data and growth and age-structure records since 1941 to 1) identify whether summer or winter climate is limiting treeline expansion at this site; and 2) determine how winter climate is affecting *Dracophyllum* growth and establishment.

## Materials and Methods

### Study area

Campbell Island (52°33.7′S, 169°09′E) is an isolated island in the southwest Pacific Ocean. The island has an oceanic climate (Conrad Index of Continentality  = −5) characterized by cool (mean annual temperature at sea level: 6.9 °C), moist (mean annual precipitation at sea level: 1380 mm), cloudy and windy conditions [20], with minimal seasonal contrast (4.2 °C mean difference between seasons). The island (*c*. 113 km^2^) is mountainous rising to 500 m elevation in the south, and dominated by low forest and scrub in the lowlands. The two dominant forest canopy species forming the treeline, *Dracophyllum longifolium* and *Dracophyllum scoparium*, are evergreen broadleaf species. Both species can reach heights up to 5 m and can reach 240 years of age on Campbell Island [5]. Hybridization is common between the species.

Although now uninhabited and having UNESCO World Heritage status, the vegetation of the island was highly disturbed by fire and farming between the time of its discovery in 1810 until the cessation of farming in 1931, but particularly during the years of farming (1895–1931) [5], [8], [19]. The effect of past land-use on stand density and age-structure was constant across the study area. Land-use affected *Dracophyllum* abundance but not distribution and the species has rapidly recolonized areas it was removed from [19]. Feral sheep remained on the island in limited locations to monitor the effects of grazing on the flora and fauna until 1987. Bestic et al. [5] found no evidence that removing grazing affected *Dracophyllum* establishment. Grazing promoted rather than hindered establishment by preferentially feeding on grasslands and herb-fields [8]. The 36-year burning and grazing period, followed by 40 years plus of grazing, provided ideal conditions for *Dracophyllum* spread unlike many treeline situations where microsite and dispersal limitations slow treeline advance. Permits to conduct data from Campbell Island were received from the New Zealand Department of Conservation. No endangered species were sampled.

### Data collection

**Figure 1:**
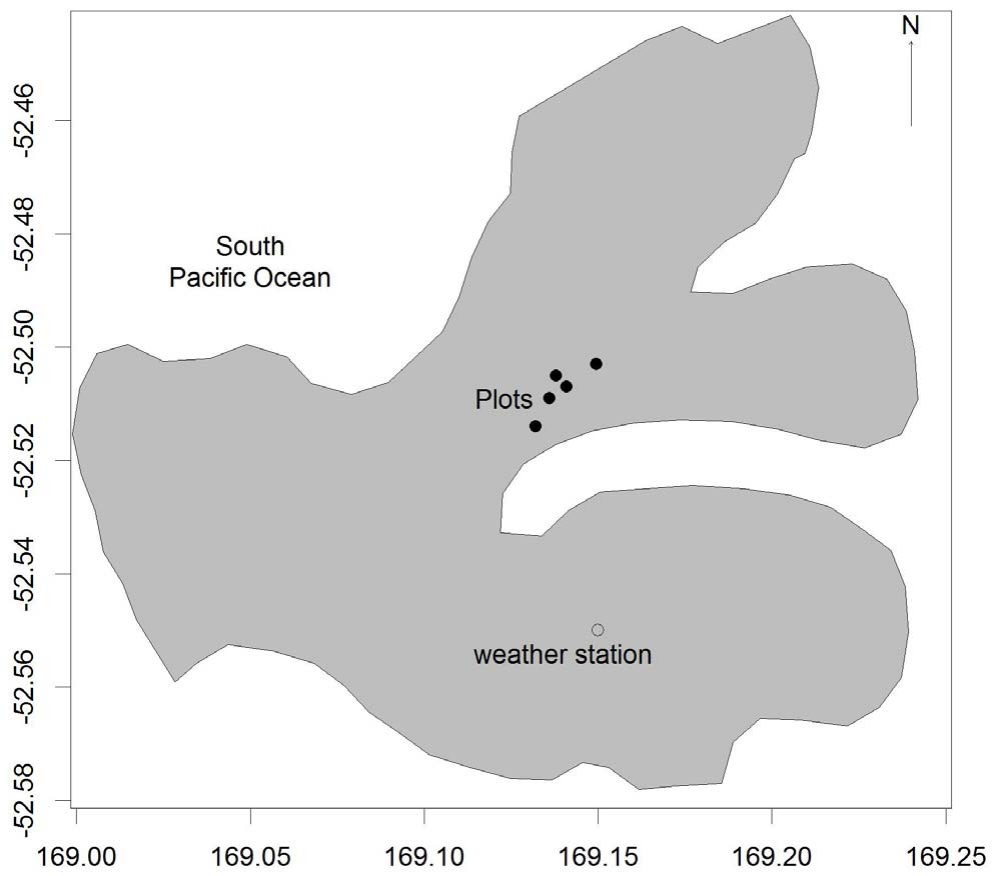
Map of plot and weather station locations on Campbell Island.

Sampling was undertaken in summer, December 1998 - January 1999. A total of 17 plots were positioned at the head of Perseverance Harbour (Fig. 1) from shoreline to shrubline (10–225 masl), spanning the variation in soil nutrient status and drainage (Table 1). Plots were classified as being mesotrophic (medium fertility and well drained) or eutrophic (high fertility and very wet, but without moving water). Soil nutrient status was assessed based on the vegetation within the plot and drainage by the degree of moisture felt when a hand was pressed firmly on to the substrate surface [21]. Sampling was conducted within circular plots, the size of which varied so as to include 15–16 individual *Dracophyllum* trees. Within each plot, a single disk was taken from all living *Dracophyllum* with diameters of at least 10 mm just above the root collar. For multi-stemmed plants the largest stem was chosen. Accumulated soil around the base of the stem was removed before sampling to ensure individual plants were sampled and to ensure samples were taken just above the root collar. Samples were limited to living stems because dead stems could not be accurately aged [21]. To take into account any bias that may be present by excluding dead stems, reconstructed age-structure was compared against estimates of *Dracophyllum* cover change developed using repeat-photography (see Wilmshurst et al. [19]). Disks were mounted, sanded and dated to reconstruct establishment patterns following standard dendrochronological procedures [22].

**Table 1:** The five plots used to assess growth-climate relationships were characterized by *Dracophyllum* species (*Dracophyllum scoparium*, *Dracophyllum longifolium*, hybrid of the two species), soil type, soil drainage, soil fertility, and elevation (masl).

Plot	Species	Soil type	Drainage	Fertility	Elevation
1	*D. scoparium*	Mesotrophic	Good	Medium	10
2	*D. scoparium*	Mesotrophic	Moderate	Medium	15
3	*D. scoparium* and hybrid	Mesotrophic	Moderate	Medium	30
4	*D. scoparium* and *D. longifolium*	Mesotrophic	Moderate	Medium	58
5	*D. scoparium* and hybrid	Eutrophic	Very poor	High	85

Soil type is based on soil drainage and fertility as defined by Bestic [21].

Analysis of growth was limited to the five plots with at least five trees aged 50 years or more. Disks were scanned at 1200 dpi and then processed using CDendro v7.3 (Cybis Elektronik and Data AB. Sweden). We counted tree ring limits and measured ring-width to 0.01 mm along four radii for each tree disk. We used the site-wide dataset for crossdating, evaluation of missing rings, and dating errors. This step was completed within CDendro v7.3, We first identified the most representative sample (base on correlation and T-test values). We then followed procedures outline in the CDendro manual to complete crossdating. In all samples, the youngest date is 1998, as all stems were living when samples were taken. We calculated the average age-detrended annual growth rates within a plot (plot-level) and across all five plots (site-level). We calculated the mean plot-level and site-level chronologies in CDendro v7.3 and then removed any age-related growth trends in the ring-width series with a smoothing “spline” with rigidity of 48 in the dplR library [23] within R v. 3.0.2 [24]. A smoothing spline is ideal in this case because we were interested in the high-frequency variation, and the method makes no assumptions of the shape of the curve to be used for standardization [25].

Our ability to detect a climate-growth signal is influenced by two factors, the location that disks were taken along tree stems (just above root collar) and the number of samples within a plot (Table S1 in File S1). Growth is influenced by factors other than climate more so at the root collar than at breast height. Thus, ability to detect a growth-climate relationship is harder using ring-widths collected at the root collar than at breast height. However, growth at the root collar is correlated with growth at breast height and year-to-year variation in growth is retained [26]. Thus, ring-width measurements taken at the root collar can provide meaningful data on climate-growth relationships. We evaluate whether growth at the root collar among trees within a plot are responding to local microscale conditions such as soil accumulation, or to conditions acting across a plot, such as climate, using Expressed Population Signal (EPS) values. EPS values are also valuable for determining whether our samples size (between 13 and 20 within a plot) is insufficient for detecting a signal. EPS quantifies how well a chronology based on a finite number of trees represents the hypothetical perfect or true chronology [27]. A low EPS value may indicate insufficient sample size or that local-level factors rather than plot-level factors are influencing ring-width. Calculated EPS values ranges from a low of 0.693 for individual plots to a high of 0.873 for the site-wide dataset. These levels indicate that we are able to detect a growth-climate signal in our data. As there was little gain between EPS values calculated at the plot and the site-wide levels, we used the plot-level data for analyses.

### Statistical analysis

Age-structure and growth records extended back to 1837. We use this full dataset to evaluate the relationship between age-structure and growth during (1837–1930) and after (1930–1993) the period of human disturbance. However, continuous climate records (temperature, precipitation) were not available prior to 1941 from Campbell Island. Temperature records before 1941 from the closest climate stations to Campbell Island were only weakly correlated with those from Campbell Island, especially prior to 1960 (Fig. S2 in File S1). We therefore limited our analysis of the relationship of growth and establishment to climate to the period in which climate records are available on Campbell Island (1941–1999). As recently established stems, those with diameters less than 10 mm, were not sampled, our analysis of age-structure trends is limited to the period 1941–1993. Austral summer is December to February inclusive; and austral winter, June-August. Explanatory variables in our growth analysis included: temperature (winter and summer, mean, maximum and minimum); temperature range (mean seasonal maximum - mean seasonal minimum); winter freezing degree days (wFDD: the summed difference in the daily mean temperature from zero over the winter months); growing degree days above 5 °C; winter and summer precipitation (identified by Wilmshurst et al. [19] as being significant on Campbell Island).

We first assessed, using Pearson's product-moment correlation, whether overall (site-wide) growth and establishment dates were correlated to the climatic variables. This was necessary to identify which variables to include in our multivariate models and was accomplished by assessing the correlation score. In our multivariate models, which evaluates the effect of climate change while taking into account the effect of plot-level variables (elevation, soil type) on growth and stage structure, we included only those climatic variables with a correlation score >0.25 and used the mean plot-level growth rates rather than the mean site-wide growth rates. Interactions between climate variables were considered but are shown only if significant.

We used logistic regression models to determine whether plot-level age-structure was associated with the explanatory variables (maximum winter temperature, winter precipitation, elevation, soil drainage, soil type, species) and linear regression models to determine whether growth rates were associated with explanatory variables (winter temperature range, winter precipitation, elevation, soil drainage, soil type, species). Age-structure, based on 646 stems across the study area, was modelled using a logistic regression model (establishment occurred or not) to take into account the high occurrence of no establishment years and the low occurrence of establishment within a plot in a given year. A zero-inflated Poisson model was considered and provided similar results to the logistic regression model when only climate was considered. This model, however, was not chosen as the model was not stable when plot data was added. Continuous explanatory variables (climatic factors, elevation) were standardized by subtracting their mean divided by their standard deviation and categorical variables (soil type, drainage, species) were included by coding these as dummy variables and choosing one of the classes as a reference class (eutrophic soil type, good soil drainage, *D. scoparium* species) with the coefficient set to zero [28].

To reduce redundancy in climatic variables, we first assessed whether climate variables were significantly correlated. If correlated, the climatic variable with the highest correlation value in the climate-growth correlation tests was included. Temporal auto-correlation was evaluated visually and statistically following Zuur et al. [29]. We found no evidence of temporal auto-correlation of climate on growth or establishment (phi <0.25). In addition, including an auto-correlation structure in the model did not affect model results. Autocorrelation was not removed from the climate data as we found no evidence of temporal auto-correlation. To assess whether growth and establishment lag climatic conditions we also ran the model, lagging growth and establishment values by one and two years (Figs S3 and S4 in File S1).

We fit the plot-level growth model in a Bayesian framework using Markov chain Monte Carlo (MCMC) methods as implemented in OpenBugs [30] called from the BRugs library in R v. 3.0.2 [24]. Non-informative prior distributions were assigned to all parameters to reflect our lack of prior information and to allow the data to drive parameter estimation. Variance parameters were assigned uniform distributions on the standard deviation in the interval 0–10 [28], while other model parameters were assigned flat normal prior distributions with mean 0 and variance 1000. The models were run with three MCMC chains and a burn-in of 10,000 iterations, which was sufficient for chains to have converged based on visual examination of the chain histories and the Gelman-Rubin statistic [31]. We continued each MCMC run for a further 10,000 iterations and used the last 10,000 iterations of each chain (30,000 iterations in total) to obtain posterior distributions for each parameter, from which we derived mean values and 95% credible intervals (parameter estimates) [28]. In Bayesian statistics credible intervals of a posterior probability distribution are used for interval estimation and are analogous to confidence intervals in frequentist statistics [28].

Following model evaluation, further analysis on how temperature range is affecting growth was necessary. Here, we used Pearson's product-moment correlation to assess the relationship between growth and the distribution of the daily maximum and minimum temperatures, both the symmetry (skew) and standard deviation (amplitude), around the annual seasonal mean maximum and minimum temperatures [32]. These two measures provide an indication of whether the effect of temperature range results from the degree of variability (standard deviation) or the occurrence of extreme values (skewed symmetry). All analyses were conducted in R v. 3.0.2 [24].

## Results

### Growth

**Figure 2:**
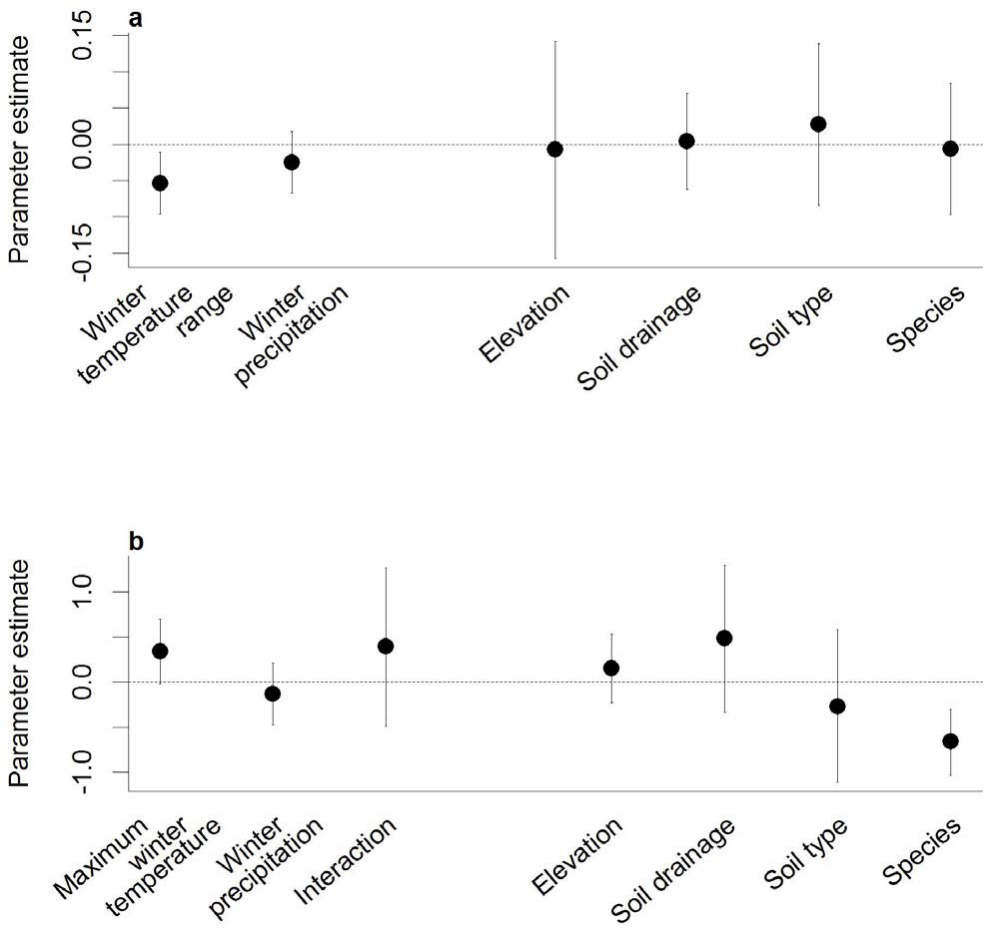
Results from the a) linear regression model for growth and b) logistic regression model for age-class structure fit within a Bayesian framework. The mean (filled dots) and 95% credible intervals (solid lines) for the parameter estimates describe the effect of each explanatory variable on plot-level annual growth rates. Categorical variables are relative to a reference class (good soil drainage, eutrophic soil type, *D. scoparium* species) as described in the Methods section. Credible intervals crossing the zero line (dashed) are not significant.

Growth rates between 1941 and 1999 were both negatively correlated with the winter temperature range and total winter precipitation (Table 2). When these two climate variables were included in the regression model with plot-level variables, only winter temperature difference was significant in explaining variability in growth rates (Fig. 2a). Growth tended to decrease with increasing winter temperature range as shown by the inverse relationship with growth in both the correlation test and regression model. An interaction between winter temperature difference and precipitation was considered but was not significant (Bayesian credible intervals overlap zero: −0.1790–0.0775).

**Table 2:** Pearson's product-moment correlations between climate variables and mean site-level annual growth and establishment between 1941 and 1999.

		Growth		Establishment		
		t-value	r	t-value	r	
Mean temperature	Summer	1.027	0.149	1.114	0.173	
	Winter	0.826	0.121	**2.608**	**0.381**	
Minimum temperature	Summer	1.516	0.218	0.751	0.118	
	Winter	1.141	0.204	**2.145**	**0.321**	
Maximum temperature	Summer	0.554	0.081	1.047	0.163	
	Winter	0.049	0.007	**2.893**	**0.416**	
Temperature range	Summer	−1.546	−0.222	0.046	0.007	
	Winter	−2.603	−0.358	0.371	0.059	
Precipitation	Summer	0.234	0.035	−0.697	−0.109	
	Winter	−1.813	−0.258	**−2.061**	**−0.310**	
GDD 5°C	Summer	1.418	0.206	0.822	0.131	
wFDD	Winter	−0.481	−0.225	**−2.122**	**−0.322**	

Relationships with a correlation score >0.25 are indicated by bold typeface. Growth and establishment values are the average values across the five plots. GGD 5 °C refers to growing degree days above 5 °C during the summer months and wFDD refers to freezing degree days calculated as the summed difference in the mean daily temperature from 0 °C over the winter months. A negative FDD value indicates temperatures above freezing (0 °C).

**Figure 3:**
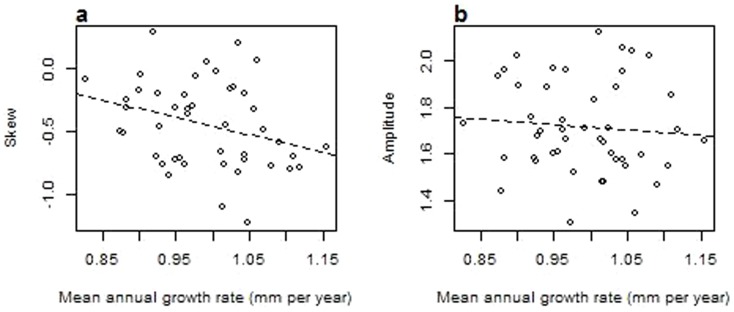
The relationship between the average site-wide mean annual growth rate and a) the symmetry of the maximum temperature around the mean (skew) and b) the amplitude of the scattering (standard deviation).

Growth was correlated (r = −0.304, p<0.05) with the symmetry of the maximum temperature around the mean (skew) but not with the amplitude of the scattering (standard deviation) or the skew and amplitude of minimum winter temperature (p>0.05; Fig. 3). In terms of skew, the majority of years exhibited a negative skew, indicating that in most years there is a decreased probability of an extremely warm winter. The negative relationship between skewness and growth indicates that frequent occurrence of above average winter warmth is associated with lower growth. We also considered the possible effects of changes in growing season length (growing degree days >5 °C) and degree of coldness (freezing degree days <0 °C). Neither growing degree days or freezing degree days were significantly correlated with growth (p>0.05; Table 2).

### Age-class structure

**Figure 4:**
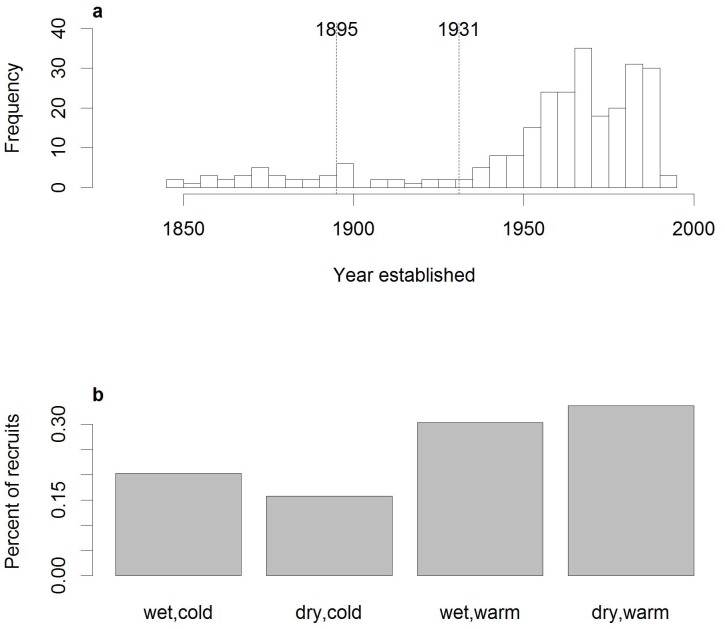
Age-class structure across all plots according to a) establishment year and b) climatic conditions. A) Frequency distributions of the year of establishment (in 5 year bins) for *Dracophyllum* plants across all plots. Dashed vertical lines represent the period in which farming occurred on the island. Livestock number and distribution were limited after 1931 but were not eradicated until 1991. This figure is modified from Bestic et al. [5]. B) The percent of total establishment across all plots in four winter climate categories between 1941 and 1993 according to winter precipitation and maximum temperature. Winter climate at the year of establishment is classed as whether winters were wet (>400 mm total precipitation) or dry (<400 mm total precipitation) and warm (>7°C on average) or cool (<7°C on average).

Periods of high establishment coincided with expansion in *Dracophyllum* cover, as recorded by repeat-photography [19], indicating that excluding dead stems did not bias our results. Humans exerted a clear influence on age-structure as establishment was low until 1930 (Fig. 4a), when farming and associated burning and wood-cutting ceased. Climate also influenced establishment between 1941 and 1993. During these 50 years, establishment was significantly correlated with winter but not summer conditions (Table 2). Establishment was positively correlated with winter temperature variables and negatively correlated with winter precipitation and winter freezing degree days (Table 2). All significant temperature variables were highly correlated (r>0.8). We therefore focus on the temperature variable most strongly correlated with establishment, the average daily maximum winter temperature and winter precipitation. When these two climate variables were included in the regression model with plot-level variables, the interaction between winter maximum temperature and winter precipitation was significant in explaining age-structure (Fig. 2b). Establishment was more likely to occur in warm, dry years than in cool or wet years (Fig. 4b). Establishment over the last 150 years is not associated with growth (r = −0.103, p = 0.226), even after taking human land-use into account (pre-human land abandonment 1837−1930, r = 0.049, p = 0.649; post-human land abandonment 1931−1983, r = −0.206, p = 0.139).

## Discussion

Both land-use legacies and climatic warming during the growing season would be expected to have positively affected growth rates and establishment at Campbell Island. Nevertheless, despite vigorous recolonization below treeline, forest and scrub have not expanded beyond the historic elevational limits of the late 19^th^ century [5], [19]. We suggest that winter climatic conditions have limited forest growth and establishment. The positive relationship between winter warmth and age-class structure is consistent with observations that warmer winters enhance establishment at treeline [33], [34]. However, our other finding, that winters with a large maximum temperature range may be detrimental to growth, is less intuitive and infrequently identified.

There are two reasons why negative effects of winter on growth have rarely been identified. First, most treeline sites are continental and have deep, persistent winter snow-packs. Once under a snow-pack, the temperature experienced by a seedling or sapling is close to zero and invariant. Winter conditions would, therefore, be expected to negligibly impact growth. However, snowpack depth and duration is declining globally with winter climate warming [17], [18]. With less snowpack, species are likely to be exposed more often to harsh winter conditions. Second, although winter warming is well-recognized, the physiological effects of winter warming are not well-studied [9]. It follows that a counter-intuitive effect, warmer winter conditions negatively affecting growth, would rarely be considered.

We explore several more likely hypothesized negative effects of winter climate. Increased precipitation in summer and winter affected both growth and forest age-class structure. The peat soils of Campbell Island exist primarily because of low evapotranspiration and persistent light rain [35] and we assume that water-logging with increased rainfall is a risk to establishment [5], [11]. However, the effect of precipitation on growth was insignificant in the linear regression model (Fig. 2a). Growth limitation resulting from winter conditions is most often thought to result from mechanical damage causing dieback [6]. Signs of dieback or damage were only rarely observed on Campbell Island, and then at exposed sites, suggesting that the effect of winter on growth is physiological rather than mechanical. Decreased snow-packs, with winter warming, could negatively affect plants through increased exposure to frost. However, snow is ephemeral on the island and no clear effect of the number of frost days or freezing degree days on growth or forest age-class structure was evident.

We propose that metabolic stress limits *Dracophyllum* growth at Campbell Island. Metabolic stress occurs when stress, whether due to climate or site-specific conditions, reduces photosynthesis more than maintenance repair (reviewed in McDowell [36]). Winter metabolic stress has been observed in both angiosperm and gymnosperm trees [14], [15], shrubs [37], and bryophytes [38] through a) direct measurements of changes in carbon reserves and root respiration [39], b) relationships between winter temperature and growth using dendroecological methods [40], and c) experimental manipulation of winter temperatures [41], [42]. Well-established research has demonstrated that roots are active during the winter months and slight changes in winter temperatures will likely affect soil processes, such as root respiration (reviewed in Crawford [14]). Roots are especially sensitive to intermittent warming as there is no photoperiodic inducement of winter dormancy or chilling requirement to resume growth [43], [44]. Winter warmth can therefore result in higher root respiration and accelerated draw-down of carbon reserves, resulting in metabolic stress and lower available carbon reserves. The effect is particularly evident at moist, high altitude sites, much like Campbell Island, presumably because temperature fluctuations have a greater impact on plants with hydrated roots [45], [46].

Metabolic stress can be expected to be low in cold winters as plants are mainly dormant, and in warm winters (mean temperatures above 5 °C), because the winter is short and the period of draw-down of carbohydrate reserves restricted. Maximum stress should occur when temperatures are frequently above average throughout the dormant period. In most years, the winter temperature range is skewed toward cooler temperatures, indicating fewer days above 5 °C and less draw down of carbon reserves. It is during years when the skew is toward warmer temperatures that growth limitation is observed. Warm temperatures in these years result in prolonged metabolic activity but insufficient nutrient availability for growth [47] or bud development [46] with subsequent exposure to cold conditions. Consistent with these expectations, we found that it was not the degree of coldness or warming during the winter but the variability and skew in the temperature range that was most strongly related to annual variation in growth (Table 2). The effect of stress on plant growth, carbon allocation, and mortality is complicated. Further field studies are necessary to test for the occurrence and long-term effect of metabolic stress.

Analysis of climate relationships to growth and age-structure were limited in this study to the last half of the 20^th^ century because of available climate records. Dendroecological records from Stewart Island, an island just south of the South Island of New Zealand, provide insight into longer-terms patterns. Stand-history reconstructions from Stewart Island were taken from the least human-impacted sites and indicate that the period of lowest establishment (1830−1920) of two evergreen conifer species coincided with a period when conditions were cool and wet. In contrast, the period with the highest establishment (1970's) corresponds with a prolonged period of dry, warm winters [48].

Our finding that winter warmth influences growth and establishment is especially significant in light of climate change. Globally, temperatures have increased over the last century and are projected to increase more rapidly during the winter months than the growing season [49], [50], [51]. In both maritime and oceanic regions, winter warming is projected to initially result in milder winters and increased establishment. Further warming, however, may lead to climatic conditions similar to those at Campbell Island at which the traditional limitations imposed by winter (extreme low temperatures, frozen ground, snow-pack, ice-blast, etc.) do not apply or only weakly. In this case, further warming may lead to decreased growth rates, even in regions where plants are currently protected from harsh winter conditions by snow cover. We expect that the negative effects of winter warming observed within the monospecific forests on Campbell Island will be less pronounced or slower to exhibit within diverse forested ecosystems with higher genetic and phenotypic plasticity than is evident within monospecific forests.

## Supporting Information

File S1 Contains the following files: **Figure S1.** Changes in the mean annual temperature (°C), mean annual precipitation (mm), and growth (mean detrended ring width across all plot) with year (1941–2000). Dashed line indicates the best fit line between year and either mean temperature, mean precipitation, or mean growth. **Figure S2.** Relationship between mean annual temperature (°C) between 1941 and 1989 at Campbell Island and a) Christchurch and b) Dunedin, the two closest mainland New Zealand climate stations with climate records extending back prior to 1940. **Figure S3.** Seasonal lag effects on growth when winter temperature range and winter precipitation are lagged by a) one and b) two years. The linear regression model was fit within a Bayesian framework specifying a lag in either one (climate: 1941–1998, growth: 1942:1999) or two years (climate: 1941–1997, growth: 1943:1999). Note that climate records were not available prior to 1941. The mean (filled dots) and 95% credible intervals (solid lines) for the parameter estimates describe the effect of each explanatory variable on plot-level annual growth rates. Categorical variables are relative to a reference class (good soil drainage, eutrophic soil type, *D. scoparium* species) as described in the Methods section. Credible intervals crossing the zero line (dashed) are not significant. **Figure S4.** Seasonal lag effects on establishment (occurred or did not) when maximum winter temperature is lagged by a) one and b) two years. The logistic regression model was fit within a Bayesian framework specifying a lag in either one (climate: 1941–1992, establishment: 1942:1993) or two years (climate: 1941–1991, establishment: 1943:1993). Note that climate records were not available prior to 1941. The mean (filled dots) and 95% credible intervals (solid lines) for the parameter estimates describe the effect of each explanatory variable on plot-level age-class structure. Categorical variables are relative to a reference class (good soil drainage, eutrophic soil type, *D. scoparium* species) as described in the Methods section. Credible intervals crossing the zero line (dashed) are not significant. **Table S1.** Summary statistics for chronologies.
(DOC)
