# Supplementary material for: Winter Climate Limits Subantarctic Low Forest Growth and Establishment
Source: PLoS One. 2014 Apr 1;9(4):e93241. doi: 10.1371/journal.pone.0093241 (PMC3972215; doi:10.1371/journal.pone.0093241)
Supplement: File S1 — Contains the following files: Figure S1. Changes in the mean annual temperature (°C), mean annual precipitation (mm), and growth (mean detrended ring width across all plot) with year (1941–2000). Dashed line indicates the best fit line between year and either mean temperature, mean precipitation, or mean growth. Figure S2. Relationship between mean annual temperature (°C) between 1941 and 1989 at Campbell Island and a) Christchurch and b) Dunedin, the two closest mainland New Zealand climate stations with climate records extending back prior to 1940. Figure S3. Seasonal lag effects on growth when winter temperature range and winter precipitation are lagged by a) one and b) two years. The linear regression model was fit within a Bayesian framework specifying a lag in either one (climate: 1941–1998, growth: 1942:1999) or two years (climate: 1941–1997, growth: 1943:1999). Note that climate records were not available prior to 1941. The mean (filled dots) and 95% credible intervals (solid lines) for the parameter estimates describe the effect of each explanatory variable on plot-level annual growth rates. Categorical variables are relative to a reference class (good soil drainage, eutrophic soil type, D. scoparium species) as described in the Methods section. Credible intervals crossing the zero line (dashed) are not significant. Figure S4. Seasonal lag effects on establishment (occurred or did not) when maximum winter temperature is lagged by a) one and b) two years. The logistic regression model was fit within a Bayesian framework specifying a lag in either one (climate: 1941–1992, establishment: 1942:1993) or two years (climate: 1941–1991, establishment: 1943:1993). Note that climate records were not available prior to 1941. The mean (filled dots) and 95% credible intervals (solid lines) for the parameter estimates describe the effect of each explanatory variable on plot-level age-class structure. Categorical variables are relative to a reference class (good soil drai [file pone.0093241.s001.doc]

Appendix A:

To infer growth- and establishment-climate relationships prior to 1941, we evaluated climate data from the two closest climate stations in New Zealand with records extending back to at least 1900, Dunedin, New Zealand (1852-), and Christchurch, New Zealand (1864-) [1] and with ENSO cycles (1876-) [2]. Although the 1941-1989 mean annual temperature on Campbell Island was correlated with the mean annual temperature at Christchurch (r = 0.556, p < 0.05) and Dunedin (r = 0.430, p < 0.05), the two climate records were poorly correlated prior to 1960 (r = 0.005, r = 0.01, respectively) (Fig. S2). Mean annual temperature on Campbell Island was not significantly correlated with the mean annual ENSO value (r = 0.031, p > 0.05).

References:

1. NIWA (2009) New Zealand’s climate is warming. Wellington, New Zealand: NIWA.

2. Bureau of Meteorology (2011) S.O.I. (Southern Oscillation Index) archives - 1876 to present. Commonwealth of Australia.

| Plot | No. of series | Mean tree diameter (cm) | Chronology Length | Ring width | Correlation*^a^* | EPS*^b^* | SD*^c^* |
| --- | --- | --- | --- | --- | --- | --- | --- |
| 1 | 13 | 6.55 + 2.32 | 160 | 1.087 | 0.333 | 0.777 | 0.319 |
| 2 | 16 | 5.92 + 1.34 | 133 | 1.018 | 0.145 | 0.771 | 0.133 |
| 3 | 20 | 3.49 + 1.32 | 66 | 1.006 | 0.219 | 0.693 | 0.139 |
| 4 | 16 | 5.82 + 1.87 | 148 | 0.979 | 0.579 | 0.873 | 0.190 |
| 5 | 15 | 1.48 + 0.64 | 47 | 1.009 | 0.205 | 0.795 | 0.294 |

Table S1: Summary statistics for the chronologies.

^a^Mean among-tree correlation.

^b^EPS, expressed population signal is a measure of chronology confidence.

^c^Among-tree strandard deviation

*
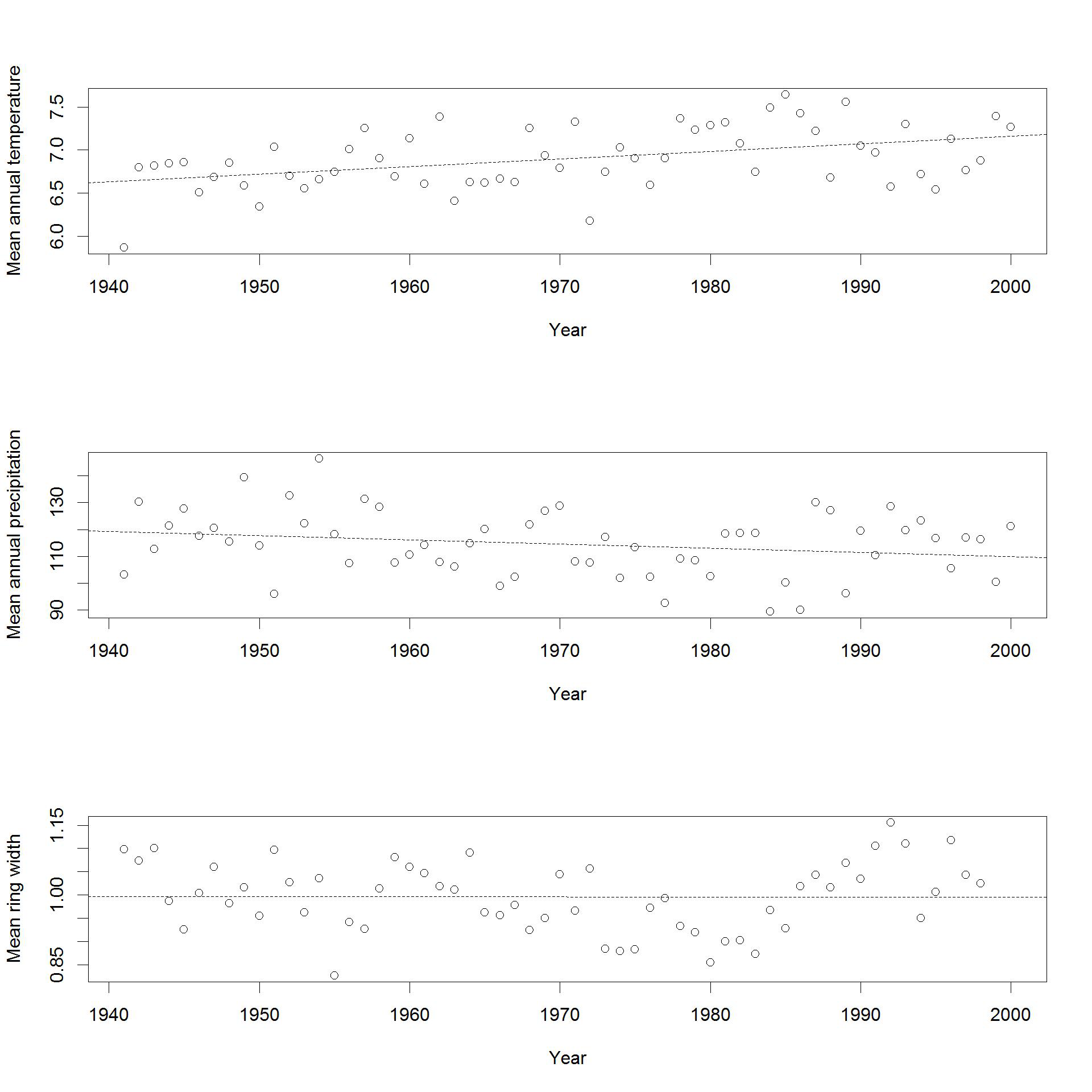
* Figure S1: Changes in the mean annual temperature (°C), mean annual precipitation (mm), and growth (mean detrended ring width across all plot) with year (1941-2000). Dashed line indicates the best fit line between year and either mean temperature, mean precipitation, or mean growth.


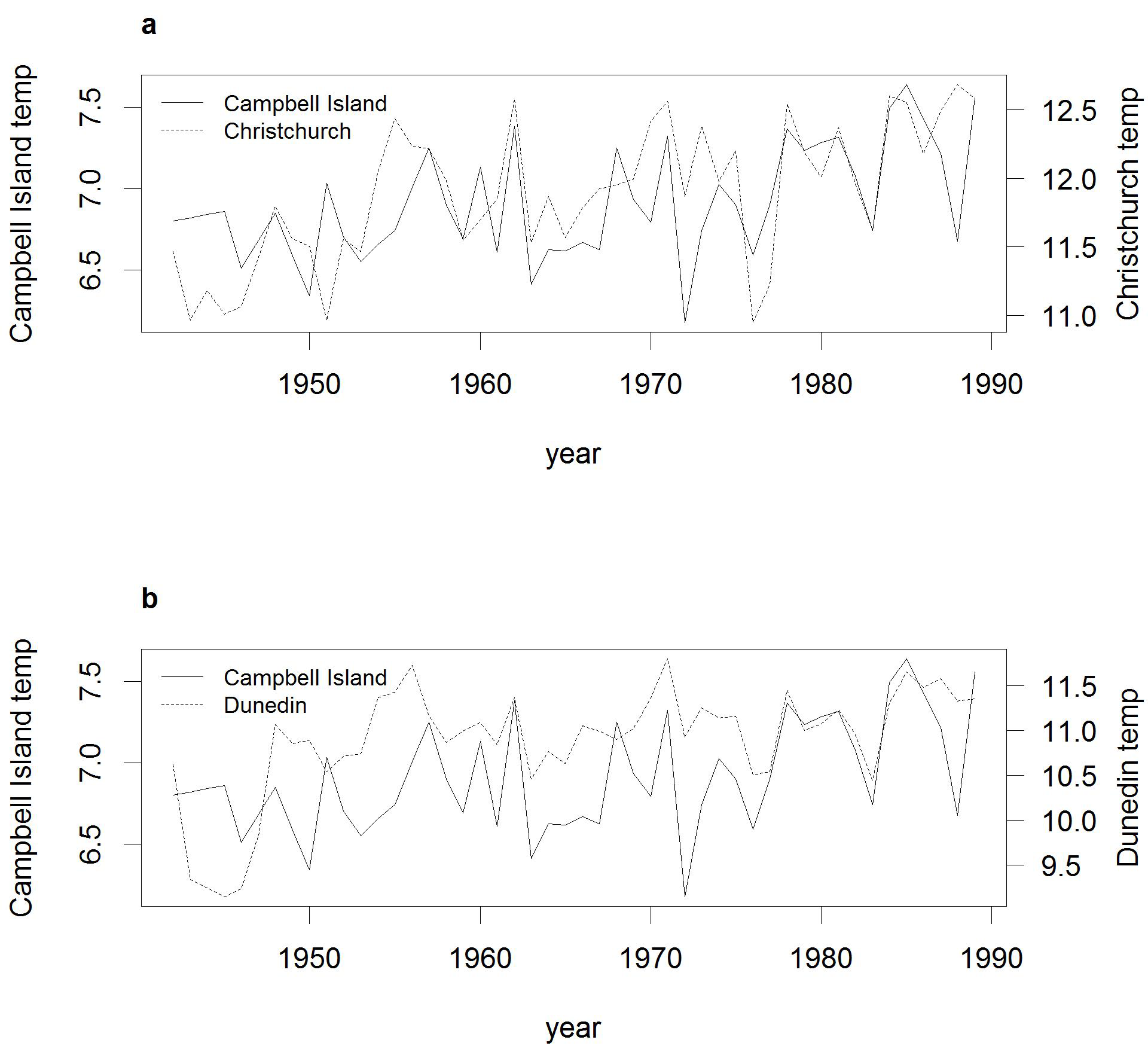


Figure S2:

Relationship between mean annual temperature (°C) between 1941 and 1989 at Campbell Island and the two closest mainland climate stations, a) Christchurch and b) Dunedin, with climate records extended back prior to 1940.


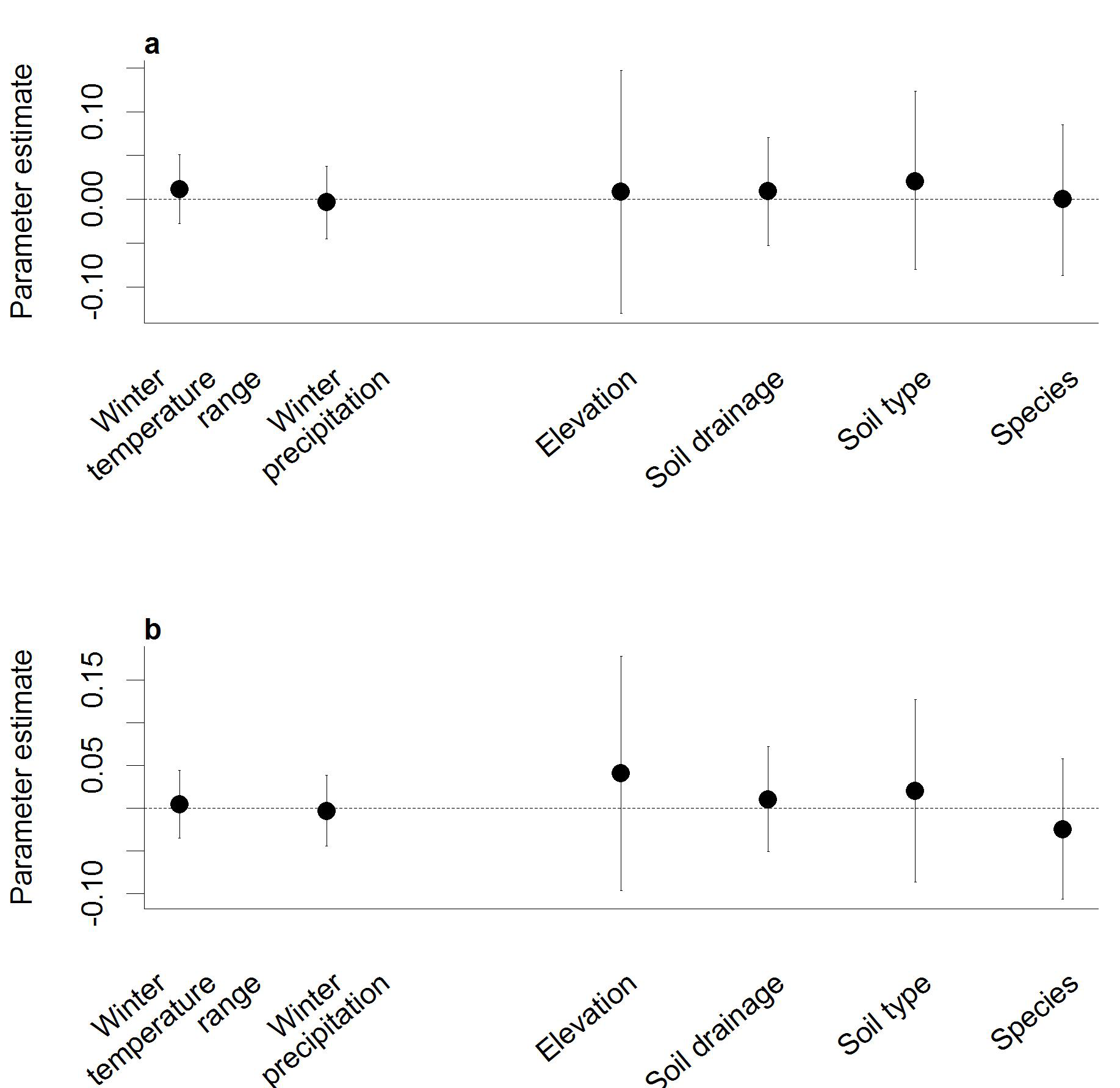


Figure S3: Effect of seasonal lag effects on growth when winter temperature range and winter precipitation are lagged by a) one and b) two years. The linear regression model was fit within a Bayesian framework specifying a lag in either one (climate: 1941-1998, growth: 1942:1999) or two years (climate: 1941-1997, growth: 1943:1999). Note that climate records were not available prior to 1941. The mean (filled dots) and 95% credible intervals (solid lines) for the parameter estimates describing the effect of each explanatory variable on plot-level annual growth rates. Categorical variables are relative to a reference class (good soil drainage, eutrophic soil type, *D. scoparium* species) as described in the Methods section. Credible intervals crossing the zero line (dashed) are not significant*.*


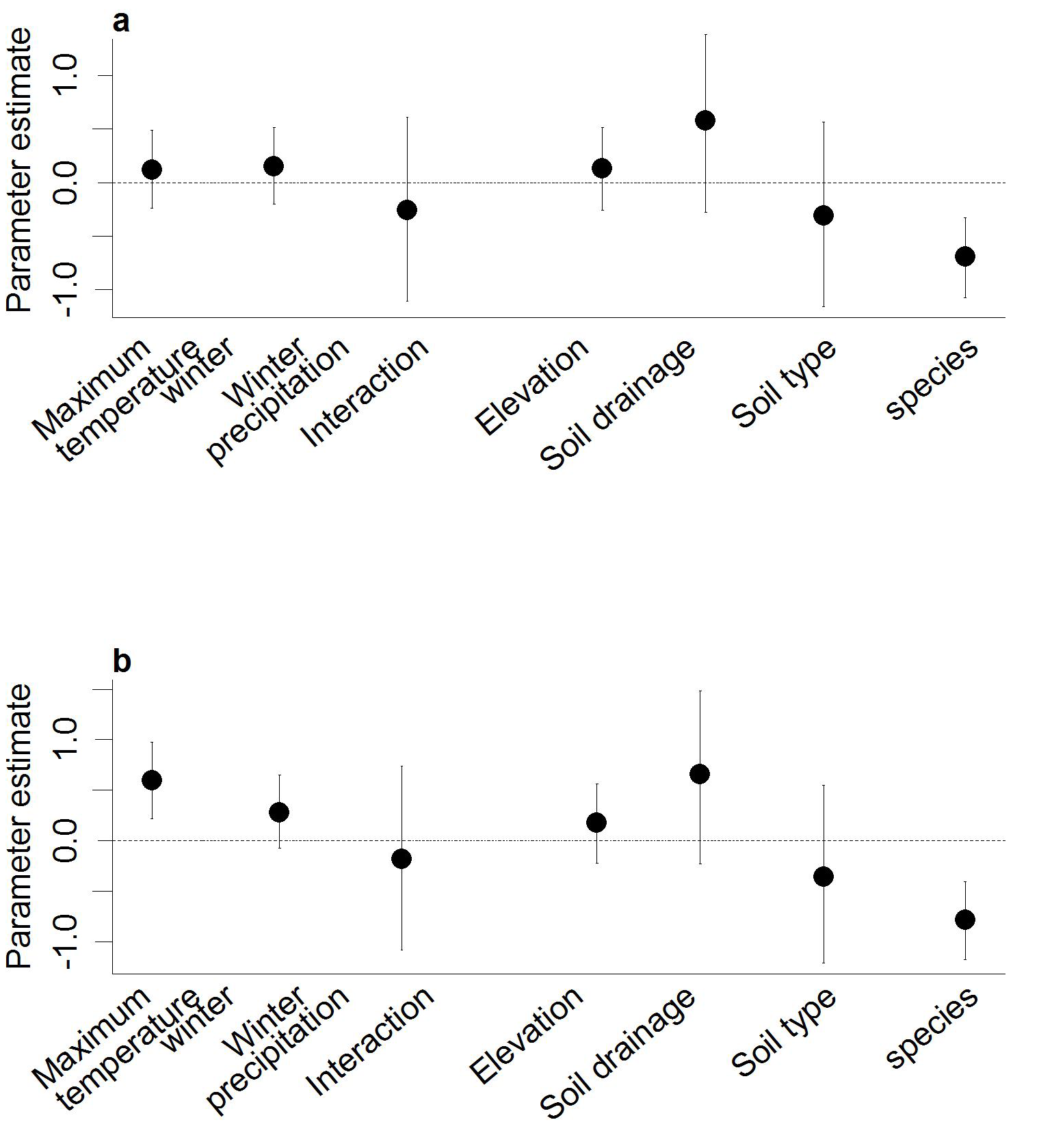


Figure S4: Effect of seasonal lag effects on recruitment (occurred or did not) when maximum winter temperature is lagged by a) one and b) two years. The logistic regression model was fit within a Bayesian framework specifying a lag in either one (climate: 1941-1992, recruitment: 1942:1993) or two years (climate: 1941-1991, recruitment: 1943:1993). Note that climate records were not available prior to 1941. The mean (filled dots) and 95% credible intervals (solid lines) for the parameter estimates describing the effect of each explanatory variable on plot-level annual recruitment occurrence. Categorical variables are relative to a reference class (good soil drainage, eutrophic soil type, *D. scoparium* species) as described in the Methods section. Credible intervals crossing the zero line (dashed) are not significant*.*
